# Supplementary material for: From creep to flow: Granular materials under cyclic shear
Source: Nat Commun. 2024 May 8;15:3866. doi: 10.1038/s41467-024-48176-6 (PMC11079021; doi:10.1038/s41467-024-48176-6)
Supplement: Supplementary file 1 — Supplementary Information [file 41467_2024_48176_MOESM1_ESM.pdf]

Supplementary Information for "From creep to flow: Granular materials under cyclic shear"

Ye Yuan[1], Zhikun Zeng[1], Yi Xing[1], Houfei Yuan[1],
Shuyang Zhang[1], Walter Kob[2,*] and Yujie Wang[1,3,4†]

[1] *School of Physics and Astronomy, Shanghai Jiao Tong University, Shanghai 200240, China*

[2] *Department of Physics, University of Montpellier and CNRS, 34095 Montpellier, France*

[3] *State Key Laboratory of Geohazard Prevention and Geoenvironment Protection, Chengdu University of Technology, Chengdu 610059, China*

[4] *Department of Physics, College of Mathematics and Physics, Chengdu University of Technology, Chengdu 610059, China*

[*] walter.kob@umontpellier.fr

[†] yujiewang@sjtu.edu.cn

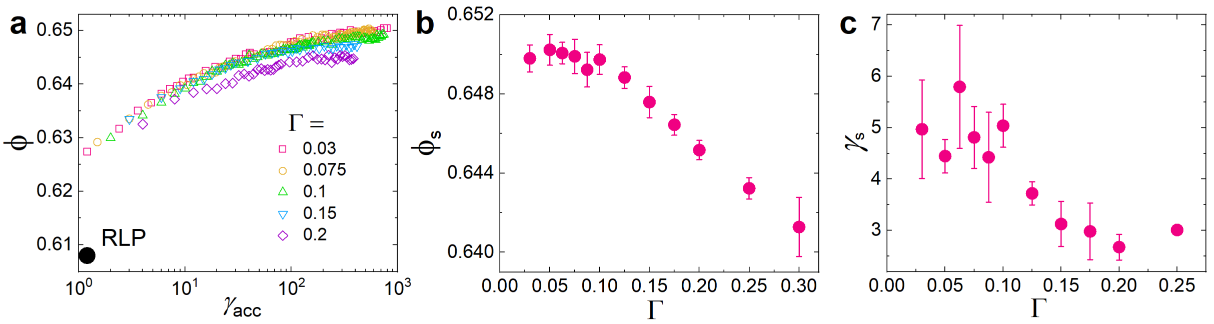

Supplementary Figure 1. **Transient process of compaction for the ABS system.** (a) Packing fraction ϕ as a function of accumulated strain γ_{acc} (defined similarly to $\Delta\gamma$ but now for a dynamics that is not in steady state) for different Γ, evolving from initially deposited packings, i.e., random loose packings (RLP, marked by black dot) with $\phi_{\mathrm{RLP}} \approx 0.608$, towards the steady state. This process can be characterized by a stretched exponential law $\phi = \phi_{\mathrm{s}} + (\phi_{\mathrm{RLP}} - \phi_{\mathrm{s}}) \exp[-(\gamma_{\mathrm{acc}}/\gamma_{\mathrm{s}})^{\beta}]$, where $\beta = 0.35$, ϕ_{s} is the steady-state packing fraction, and γ_{s} is the compaction strain scale. (b) and (c) show, respectively, ϕ_{s} and γ_{s} versus Γ and one observes a crossover at $\Gamma \approx 0.1$. Error bars represent the standard deviations from different realizations.

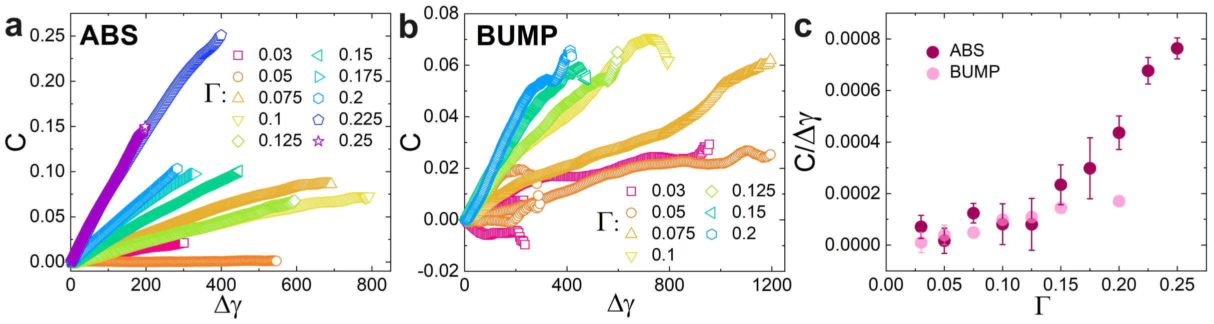

Supplementary Figure 2. **Convection strength in steady state for both systems.** (a) and (b): Up/down asymmetry in the particle motion in the two systems, characterized by $C(\Delta\gamma) = \langle\delta z\rangle/\sqrt{\langle\delta x^2(\Delta\gamma)\rangle}$, as a function of $\Delta\gamma$. C grows linearly with $\Delta\gamma$ with slopes that depend on Γ, indicating an upward motion, i.e., convection. (c) The associated slope $C/\Delta\gamma$ as a function of Γ shows a crossover at $\Gamma \approx 0.1$ for the ABS system. This is further evidence that the dynamics changes at $\Gamma \approx 0.1$ for ABS, allowing the convection to set in for large Γ. Error bars represent the standard deviations from different realizations.

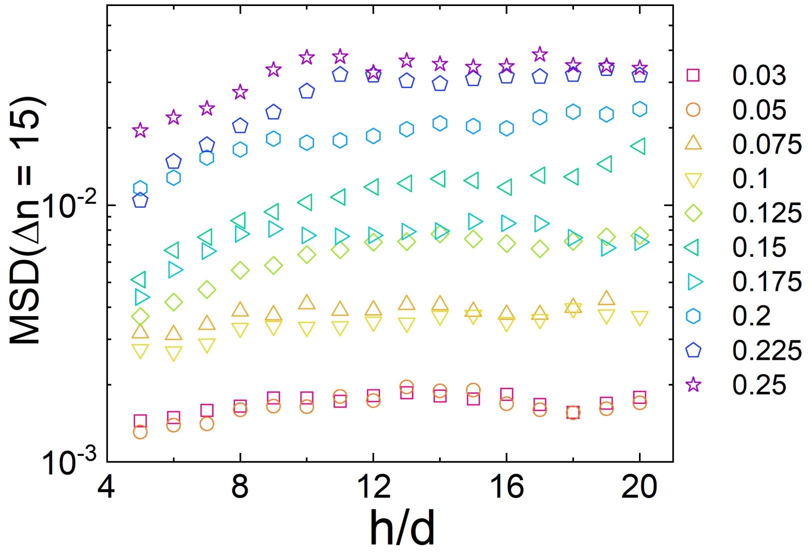

Supplementary Figure 3. **Height profile of MSD at $\Delta n = 15$ for the ABS system.** h/d is the height from the cell bottom normalized by the small particle diameter, which starts from $h/d = 4$ since we exclude the particles at the boundary. One finds only a very mild height dependence of the dynamics, in contrast to the clear Γ-dependence.

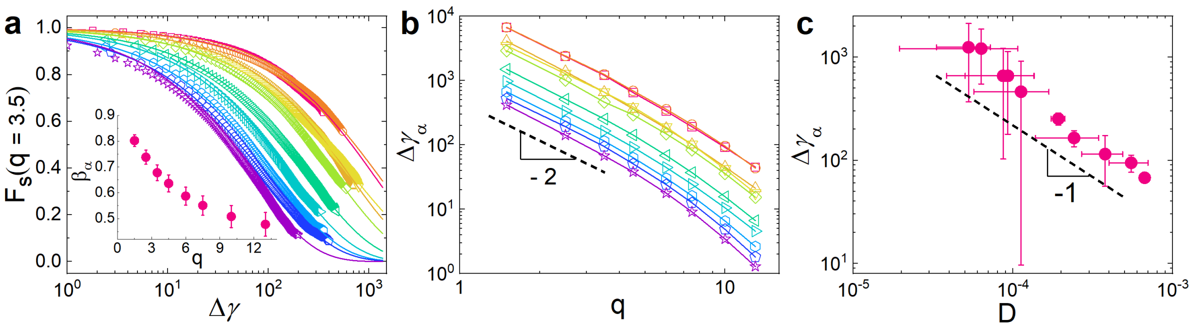

Supplementary Figure 4. **Self-intermediate scattering function $F_s(q, \Delta\gamma)$ for the ABS system.** (a) $F_s(q, \Delta\gamma)$ for $q = 3.5$, i.e., the first peak in the static structure factor, and different Γ. Solid curves indicate a stretched exponential fit $F_s(q, \Delta\gamma) = \exp[-(\Delta\gamma/\Delta\gamma_\alpha)^{\beta_\alpha}]$, where $\Delta\gamma_\alpha$ is the relaxation strain scale. Inset: Stretching exponent β_α as a function of q. (b) $\Delta\gamma_\alpha$ as a function of q for different Γ. The scaling $\Delta\gamma_\alpha \propto q^{-2}$ for small q (dashed line) indicates the Gaussian dynamics for large length scale, in agreement with the limit $\beta_\alpha \to 1$ for $q \to 0$ shown in the inset of panel (a). The color codes in (a) and (b) are the same as in Supplementary Fig. 2(a). (c) $\Delta\gamma_\alpha \propto D^{-1}$ (dashed line) for $q = 3.5$ and different Γ, indicating that the relaxation of $F_s(q, \Delta\gamma)$ conveys similar information as the MSD presented in the main text. Error bars represent the standard deviations from different realizations.

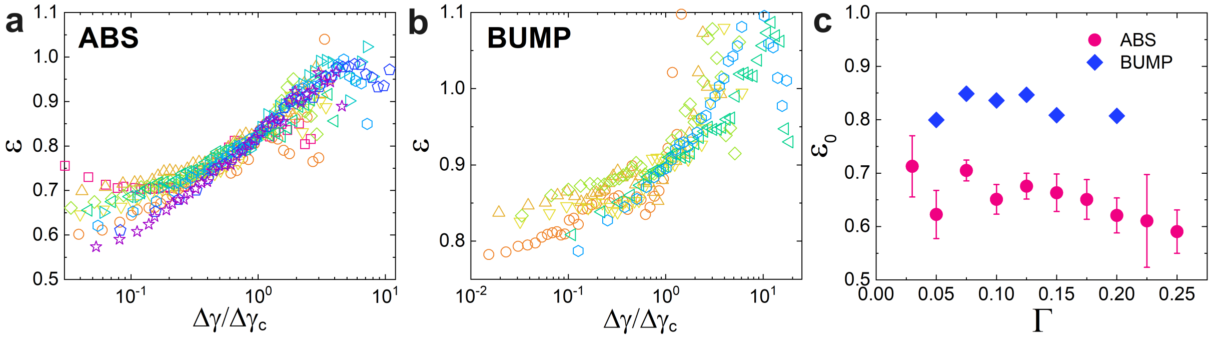

Supplementary Figure 5. **Scaling of the mean squared displacement to determine the yielding strain $\Delta\gamma_c$ for both systems.** (a) and (b): Local power-law exponent $\varepsilon = d\log(\langle\delta x^2\rangle)/d\log(\Delta\gamma)$ as a function of $\Delta\gamma/\Delta\gamma_c$ for the ABS and BUMP systems, respectively. The color codes are respectively the same as in Figs. 2(a) and (b) of the main text. The yielding strain $\Delta\gamma_c$ is defined by locating the crossover point $\varepsilon = 0.825$ (or 0.9) for ABS (or BUMP), i.e., a value in the middle between the sub-diffusive exponent at small strain and 1.0 in the large strain limit. (c) The estimated sub-diffusive exponents from averaging $\varepsilon(\Delta\gamma/\Delta\gamma_c \lesssim 0.1)$ for both systems, which confirms that they are independent of Γ. Error bars represent the standard deviations from different realizations for the ABS system.

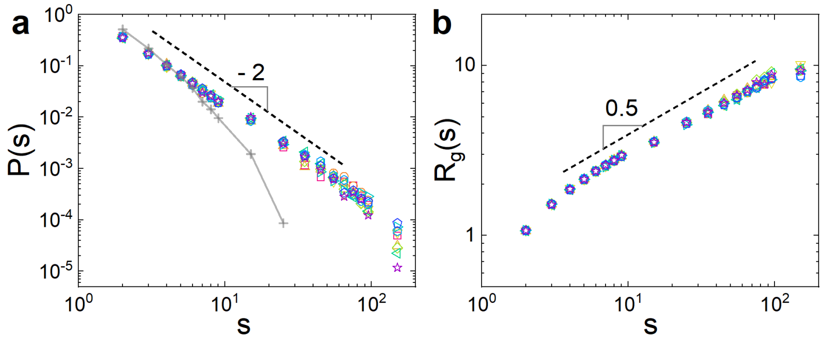

Supplementary Figure 6. **Cluster characterization of the most mobile particles for the ABS system.** We apply the same criterion as in Fig. 4(c) (see main text) to define clusters of the top 10% fastest particles. Color codes are the same as in Supplementary Fig. 2(a). (a) Cluster size distribution $P(s)$ for different Γ at the yielding point, i.e., $\Delta\gamma = \Delta\gamma_c$. The dashed line indicates an approximately universal scaling $P(s) \propto s^{-2}$. $P(s)$ from the randomly chosen 10% (line with crosses) deviates strongly from this master curve. (b) Also the average radius of gyration $R_g(s)$ of the clusters as a function of s shows a universal scaling $R_g(s) \propto s^{0.5}$ (dashed line).

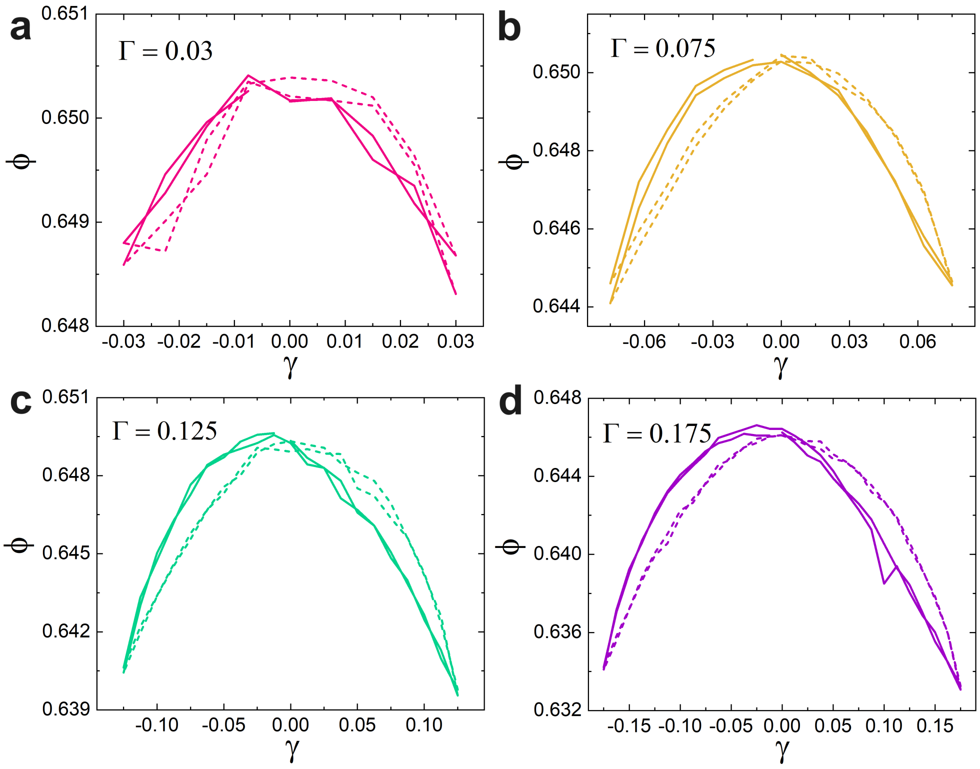

Supplementary Figure 7. **Evolution of the packing fraction during the shear cycle for the ABS system.** Data for $\Gamma = 0.03$, 0.075, 0.125, and 0.175 are shown in (a-d), and each panel covers two consecutive cycles after the system has reached the steady state. For a better visualization, we use a solid curve for the forward direction of shear (increasing γ) and a dashed curve for the backward direction. The persistent hysteresis found here resembles the findings of Ref. [45], indicating the absence of an elastic regime.
